# Pediatric delamanid treatment for children with rifampicin-resistant TB

**DOI:** 10.5588/ijtld.22.0264

**Published:** 2022-10-01

**Authors:** N. Tyeku, I. Apolisi, J. Daniels, B. Beko, B. Memani, L. Cengani, S. Fatshe, N. Gumede, K. Joseph, S. Mathee, J. Furin, C. Maugans, H. Cox, A Reuter

**Affiliations:** 1 Médecins Sans Frontières, Khayelitsha, Cape Town, South Africa; 2 Department of Health, Province of the Western Cape, Cape Town, South Africa; 3 Department of Health, City of Cape Town, Cape Town, South Africa; 4 Department of Global Health and Social Medicine, Harvard Medical School, Boston, MA, USA; 5 The Sentinel Project on Pediatric Drug Resistant Tuberculosis, Boston, MA, USA; 6 Institute of Infectious Diseases and Molecular Medicine, Division of Medical Microbiology, University of Cape Town, Cape Town, South Africa

Dear Editor, Children are a vulnerable population when it comes to TB, including rifampicin-resistant forms of the disease (RR-TB).^1^ Although the largest challenge facing children with RR-TB is being diagnosed and started on appropriate treatment, they also face a difficult treatment journey.^2^ Children with RR-TB are reported to have excellent treatment outcomes,^3^ but treatment is characterized by highly centralized services and limited pediatric formulations of second-line drugs.^4^ This complicates the care of very young children, as adult tablets need to be cut, crushed and mixed prior to administration. These manipulations often require hospitalization for the duration of therapy, with all the related difficulties for young patients and their families.^5^ Delamanid (DLM) is a newer, nitroimidazole agent developed for the treatment of RR-TB and first recommended by the WHO in 2013.^6^ The medication development plan included a robust pediatric component, and thus DLM was the first novel TB drug specifically recommended for children as part of all-oral therapy.^7^ Concerns about manipulating the adult tablet led to impractical recommendations regarding DLM use in young children, in which the drug was theoretically recommended but the weight-based dosing could not be achieved with the adult formulation.^8^ The 25 mg pediatric formulation of DLM that was tested in randomized clinical trials has only recently became available, and experience with its use under program conditions is limited.

We report here on a cohort of eight children who received treatment with the new pediatric formulation of DLM as part of a community-based program in the peri-urban township of Khayelitsha in Cape Town, South Africa. Khayelitsha is home to about 500,000 individuals and is a high-burden HIV and RR-TB setting, with approximately 200 individuals diagnosed with RR-TB each year. Since 2019, the Departments of Health from the Province of the Western Cape and the City of Cape Town have been working in partnership with Médecins Sans Frontières (MSF) to provide community-based diagnostic, preventive and treatment services to children exposed to RR-TB.^9^ As part of this program, children diagnosed with RR-TB are treated through one of 10 primary care clinics in the catchment area if they are clinically stable, or they are referred for hospitalization if they have a clinical indication. Children are treated with all-oral regimens either based on their own drug susceptibility test results or on those of their close household members who were living with RRTB. Regimens are designed following South African national guidelines for the treatment of RR-TB,^10^ and include a minimum of four anti-TB medications given for a period of 9–18 months, depending on disease severity. Children between the ages of 3 and 6 years were preferentially treated with DLM using weight-based dosing. The pediatric formulation for DLM was obtained through a named-patient-basis administered by Otsuka Pharma, Tokyo, Japan (for more information on this program, please contact medical@otsuka-onpg.com). Children in need of the pediatric formulation of DLM (a 25 mg, dispersible, mango-flavored tablet) were assessed by the medical team in Khayelitsha and then reviewed by a team of external clinical experts at the Sentinel Project on Pediatric Drug-Resistant Tuberculosis.^11^ Following this, a regimen was proposed and a formal application submitted to Otsuka for the pediatric formulation. Approval to import the medication was obtained via the South Africa Health Products Regulatory Authority using their section 21 mechanism (https://s21portal.sahpra.org.za/). DLM was administered as part of a combination regimen usually for 24 weeks. Weight-based dosing was used for all components of the regimen, including DLM, following South African guidelines. Caregivers prepared and administered all the medication after counseling by healthcare providers. This study was approved by the University of Cape Town Human Research Ethics Committee, Cape Town, South Africa (HREC 499/2011).

Between February 1, 2021 and February 28, 2022, eight children aged ≤6 years were deemed eligible for treatment with the pediatric formulation of DLM. Applications were submitted for all eight children, and all were approved for pediatric DLM. The turnaround time for submission of the applications to Otsuka was less than 2 weeks, and the medications were shipped and arrived for treatment in Khayelitsha within 10 days of approval. Thus, the overall turnaround time from application to receipt of the medication was less than 4 weeks. While awaiting the medication, adult tablets of DLM were given in the primary care clinics. At the time of writing, four of the eight children have successfully completed treatment and are clinically cured. Three of the children are responding to treatment clinically and are likely to be cured, and one child was only recently initiated on treatment. The pediatric formulation was well-liked by all eight children and their caregivers – it was easy to prepare and administer and had a pleasant taste. Two of the eight (25%) had their DLM stopped prior to 24 weeks because they developed Grade 1 and Grade 3 hallucinations and nightmares,^12^ a newly described adverse event associated with DLM use. In one child, these were recurrent visual hallucinations and greatly disrupted her sleep, but in the second child, there was just one single occurrence (see Table for more details). Isolated nightmare events can be common in young children and may not be an indication of DLM toxicity. Both children were also receiving terizidone as part of their combination therapy, another medication with well-described neuropsychiatric adverse events. One child was also receiving isoniazid another medication that can have central nervous system effects. Both children had complete resolution of these symptoms with discontinuation of DLM. None of the eight children experienced QTcF prolongation. The Table summarizes the demographic and clinical characteristics of the cohort of children receiving the pediatric formulation of DLM.

**Table:** Demographic and clinical summary of children treated with pediatric DLM

ID no	Age at treatment start (years)	Gender	Weight at treatment start (kg)	Disease characteristics	Treatment regimen and duration	Outcome	DLM-related adverse events (graded according to CTCAE version 5)	Comments
1	4	M	15.8	Clinically diagnosed, non-severe, pulmonary TB	DLM-CFZ-LVX-TRD 12 months	Treatment successfully completed	None	
2	5	F	17.5	Clinically diagnosed, non-severe, pulmonary TB	DLM-CFZ-LVX-TRD-INH 12 months	Treatment successfully completed	Grade 1 hallucinations Hallucination occurred one time 3 weeks after the drug was started and was described after evening dose as seeing a fish jump out of water onto the bed	No substitute as mild disease, contact RR-TB
3	3	F	13.6	Clinically diagnosed, non-severe, pulmonary TB	DLM-CFZ-LVX-TRD 12 months	Treatment successfully completed	None	
4	4	F	13	Bacteriologic confirmation of RR-TB, non-severe, pulmonary TB	DLM-CFZ-LVX-TRD-INH^H^ 12 months	Treatment successfully completed	None	
5	4	F	14	Clinically diagnosed, severe, pulmonary TB	DLM-LZD-CFZ-TRD 18–24 months	On treatment	Grade 3 hallucinations Hallucinations were recurrent over a 2-week period and began 4 weeks after drug started. They were described as seeing a person present who was not actually there. They interfered with sleep. They occurred more often if the medication was taken just before bedtime, rather than after dinner. Because they were recurrent and interfered with sleep, they were given a CTCAE grade of 3	ETH added to replace DLM and BDQ later added to regimen Contact with person with FQ-resistant TB
6	6	M	21	Clinically diagnosed, non-severe, pulmonary TB	DLM-BDQ-LZD-CFZ-TRD 18–24 months	On treatment	None	Contact with MDR-TB and FQ-resistant TB
7	5	M	12.2	Clinically diagnosed, non-severe, pulmonary TB	DLM-CFZ-LVX-TRD 12 months	On treatment	None	
8	1	F	9.8	Clinically diagnosed, non-severe, pulmonary TB	DLM-CFZ-LVX-TRD 12 months	On treatment	None	

DLM = delamanid; CTCAE = Common Terminology Criteria for Adverse Events; CFZ = clofazimine; LVX = levofloxacin; TRD = terizidone; INH = isoniazid (10 mg/kg/day); RR-TB = rifampicin-resistant; INH^H^ = high-dose INH (15–20 mg/kg/day); LZD =linezolid; ETH = ethionamide; BDQ = bedaquiline; FQ = fluoroquinolone; MDRTB = multidrug-resistant TB.

We report here primary care-based experience with the pediatric formulation of DLM for children treated for RR-TB in the community setting of Khayelitsha. Overall, the formulation was well accepted and tolerated, although two children stopped DLM before the prescribed 24 weeks. Children with RR-TB need to be able to receive this DLM formulation under programmatic ambulatory conditions and access should increase because the drug is now available from the Stop TB Partnership’s Global Drug Facility.^13^ Broader access to child-friendly formulations of all second-line drugs is an urgent priority in pediatric RR-TB, and countries need to prioritize the procurement of existing pediatric formulations of these treatments for vulnerable children. These formulations are an important part of the decentralized pediatric TB services that have recently been recommended by the WHO.^14^ Our experience using pediatric DLM shows that child-friendly medications are an essential part of providing decentralized, person-centered care for children with RR-TB and their families.
